# Respiratory Physiology on a Chip

**DOI:** 10.6064/2012/364054

**Published:** 2012-07-08

**Authors:** Sanjeev Kumar Mahto, Janna Tenenbaum-Katan, Josué Sznitman

**Affiliations:** Department of Biomedical Engineering, Technion-Israel Institute of Technology, Haifa 32000, Israel

## Abstract

Our current understanding of respiratory physiology and pathophysiological mechanisms of lung diseases is often limited by challenges in developing *in vitro* models faithful to the respiratory environment, both in cellular structure and physiological function. The recent establishment and adaptation of microfluidic-based *in vitro* devices (*μ*FIVDs) of lung airways have enabled a wide range of developments in modern respiratory physiology. In this paper, we address recent efforts over the past decade aimed at advancing *in vitro* models of lung structure and airways using microfluidic technology and discuss their applications. We specifically focus on *μ*FIVDs covering four major areas of respiratory physiology, namely, artificial lungs (AL), the air-liquid interface (ALI), liquid plugs and cellular injury, and the alveolar-capillary barrier (ACB).

## 1. Introduction

Recreating realistic features of the lung environment within an experimental model system is among the great challenges of modern respiratory physiology. The experimental task at hand is rendered prohibitively difficult due to limitations in controlling physiological functions, maintaining differentiation and expression of tissue-specific cellular functions, preserving the homeostatic cellular microenvironment, and integrating alveolar-capillary barrier, to name a few challenges. Although *in vivo* and *ex vivo* approaches have been successful at uncovering various aspects of pulmonary physiology, major concerns revolve around the source and supply of human lung tissue [1]. Recently, microfluidic devices have shown tremendous potential in developing alternative approaches to *in vitro* models for biological and pharmaceutical applications [2]. In particular, microfluidic-based *in vitro* devices (*μ*FIVDs) offer several advantages over conventional *in vitro* models, and, as a result, this sophisticated miniaturized technology has been adapted to *in vitro* lung modules as well [2]. The specific advantages and drawbacks of *μ*FIVDs for respiratory physiology applications are summarized in Table 1. For a more detailed discussion on other types of *μ*FIVDs, readers are invited to consult pertinent reviews on the topic [3–5].

**Figure 1:**
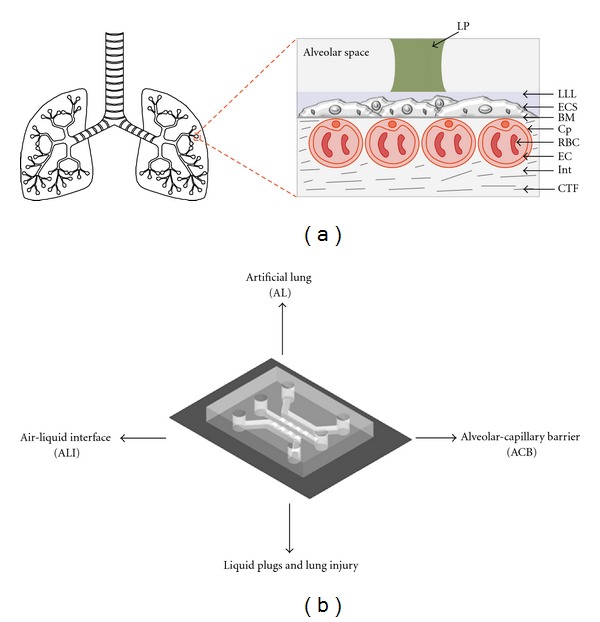
(a) Schematic of human lung structure showing an enlarged view of an alveolar-capillary barrier (ACB) in a pulmonary alveolus. Apical side of the epithelium cell sheet (ECS) lying on a basement membrane (BM) is covered with a thin lung liquid lining (LLL) layer and forms an air-liquid interface (ALI). Instabilities at the ALI can create a liquid plug (LP) that occludes small airways. The basal side of the barrier is made of an interstitium (Int) and capillaries (Cp) in close juxtaposition with the BM. Capillaries possess an inner lining of endothelial cells (ECs) and red blood cells (RBCs) flow through these vessels. The interstitium contains connective tissue fibers (CTFs) and other various types of cells. (b) Application of microfluidic technology across four major areas of respiratory physiology. The simplest microfluidic device is generally fabricated by sealing a poly(dimethylsiloxane) (PDMS) mold against a flat substrate (e.g., glass and PDMS).

The aim of the present paper is to address recent progress undertaken in respiratory physiology using microfluidic technology. We discuss, in particular, recent *μ*FIVDs developed over the past decade that mimic highly intricate lung structures (Figure 1(a)) and their ensuing complex physiological functions. Conventional approaches and their limitations are also briefly discussed. Here, we consider four highly relevant areas of modern respiratory physiology (Figure 1(b)) and highlight current efforts being put forward to advanced *in vitro* methods for understanding pathophysiology of human lung injury and diseases.

## 2. Artificial Lungs

The human lung is a remarkable organ optimized for respiratory gas exchange. Until present day, man-made engineered devices have generally failed to achieve the extraordinary efficiency of this delicate structure. The alveolar-capillary interface brings air and blood into intimate contact (Figure 1(a)), and the total area available for gas exchange is comparable to the size of a tennis court, that is, on the order of 100 m^2^ [6]. The extremely dense arrangement of the alveolar-capillary structure creates a surface area to blood volume ratio of 300 cm^−1^ [7], which maximizes the efficiency of gas exchange rate. In particular, the combination of a large surface area with a thin air-blood barrier (<1 *μ*m) [8] meets the significant requirements needed for oxygen exchange rates, varying from 200 mL/min of ambient air at rest to 5 L/min under exercise conditions [7]. Insufficiency or disorders in pulmonary functions, along with cardiopulmonary interventions, often require artificial respiratory support. Moreover, a need for an advanced artificial lung (AL) arises as a bridge-to-transplant, that is, during the waiting period prior to lung replacement surgery. Indeed, patients with severe respiratory disabilities that require whole lung transplant must usually wait for over a year until a donor is found [9]. For such cases, a long-term solution is needed: a stable and permanent AL that can improve life quality and reduce mortality while awaiting transplant [10, 11].

Artificial lungs are designed to compensate for insufficient respiratory functions of the natural lungs. In other words, these medical devices are required to oxygenate blood and simultaneously remove carbon dioxide. Traditionally, ALs are constructed from cylindrical bundles of hydrophobic hollow fiber membranes packaged in an extracorporal device. For a detailed discussion on the basic principles and traditional engineering approaches to ALs, the reader is referred to detailed reviews on the subject [7, 11]. Briefly, oxygen flows on the luminal (inner) side of the bundles, while blood flows through the device, that is, around the fibers. Under such configuration, diffusion-driven gas exchange occurs: oxygen propagates down a partial pressure gradient into the blood stream while carbon dioxide transfers into the fiber lumen. Unfortunately, such designs generally exhibit long diffusion distances (i.e., a membrane thickness is typically 10–30 *μ*m) together with a low surface area to blood volume ratio (~28 cm^−1^) and hence fail to replicate features of the native lung. The result, as expected, is a low gas exchange rate that hardly matches the requirements for resting metabolic needs, even when supplying 100% oxygen gas through the fibers. Other limitations also jeopardize the efficiency of fiber-based designs. To begin, although fibers are hydrophobic, plasma leakage occurs over time. In turn, protein and phospholipidic clots are created within the device leading to a decrease in gas exchange efficiency [12]. In addition, blood flow pathways within some AL designs are nonphysiological; they feature relatively high shear forces upon flowing blood that are hard to predict or control. These forces can cause damage to red blood cells (RBCs) such as platelet activation, stimulate inflammatory responses, and give rise to blood clotting, all of which result in higher morbidity and mortality rates [10, 12]. Finally, thrombus formation requires anticoagulant administration which can cause bleeding and electrolyte-related imbalance that call for additional medical care [12]. Overall, the leading disadvantages of a traditional artificial lung include (Table 1) (i) low gas exchange rates due to geometry restrictions of the porous membranes, (ii) short lifespan as a result of materials used in the devices, and (iii) restricted mobility for patients with the requirement for 100% oxygen gas delivered through pressured gas containers.

**Figure 2:**
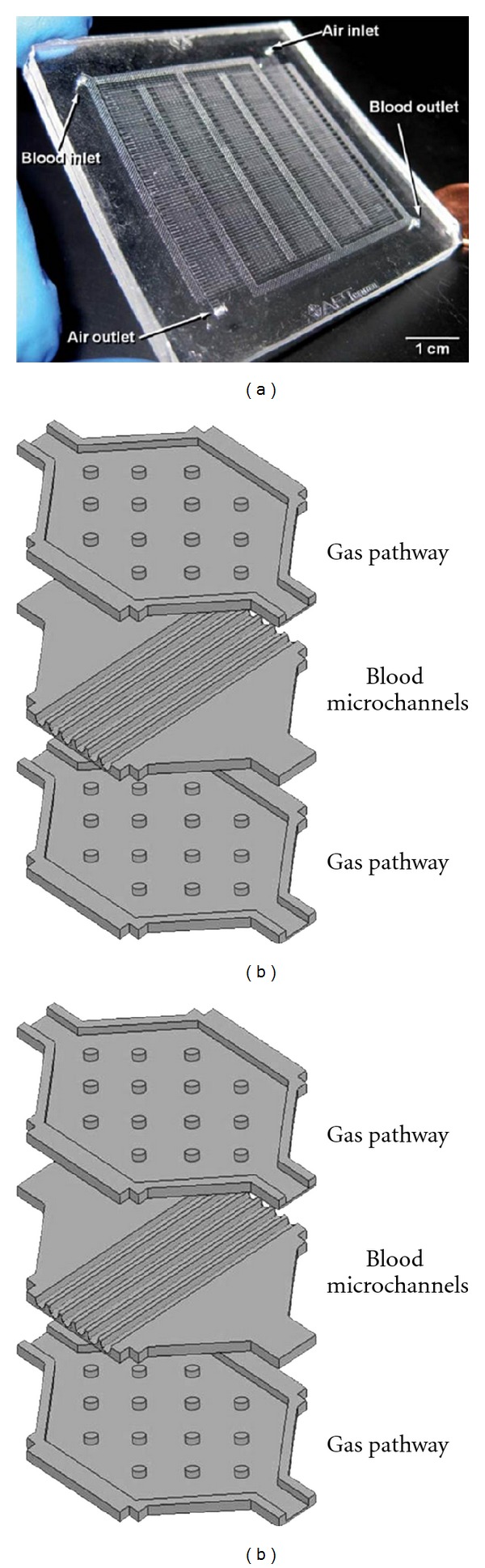
An example of a *μ*FIVD of an artificial lung (AL). A microfluidic device mimicking physiological functions of the native lung. Air and blood inlets are shown and branched architecture of microchannels can be noted. Reproduced from Potkay et al. [9] with permission from The Royal Society of Chemistry.

In recent years, *μ*FIVDs have become attractive platforms in an effort to design an artificial lung assist (Figure 2). In particular, microfluidic systems yield compact devices with micron-size channel diameters and larger surface area to volume ratio, approximating more closely features of the natural lung. Moreover, microfluidic systems enable culturing cellular monolayers within the microchannels to create a biocompatible environment for blood flow. Since endothelial cells are known to secrete anticoagulant factors *in vivo*, coating microchannels with endothelium might improve biocompatibility and diminish thrombosis occurrence, ultimately reducing the necessity for systemic anticoagulant administration. The feasibility of including such endothelial monolayers in microfluidic AL devices was recently demonstrated [13]. Microfabricated systems also allow for easier prediction and control over flow parameters, including shear stresses created during blood flow within the device. Indeed, computational fluid dynamics (CFDs) are commonly used to carefully design the delicate architecture of a microfluidic AL device [9, 10, 12, 14].

One of the first groups to utilize *μ*FIVDs towards developing an AL was Burgess et al. [13] who fabricated a small three-dimensional (3D) module of poly(dimethylsiloxane) (PDMS). PDMS, a widely used polymer for microfluidics [15], is a favorable material for ALs. Among its advantages are its high permeability to gas, satisfactory performance with blood-contact applications, and cost effectiveness [12]. Each module contained up to six alternating PDMS layers (<100 *μ*m thick) of either blood microchannels or gas pathways stacked on top of each other. This microfluidic device featured straight channels for oxygen and blood pathways and provided a surface area to blood volume ratio of 1000 cm^−1^. However, in order to achieve the gas exchange rate required for resting conditions, around 600,000 channels would have to be incorporated within a single device. Another possible reason for rather low gas exchange rates stems from the large diffusion distances; since gas exchange in the device occurs through the alternating layers, thickness of the layers is a limiting factor for gas permeance. Indeed, gas permeability was shown to decrease with increasing layer thickness [12, 13]. In general, permeability of the membrane for O_2_ and CO_2_ remains critically important, as is impermeability to fluids. To satisfy both requirements, ongoing efforts are invested to optimize and improve gas exchange membranes in microfluidic ALs [16].

By mimicking the highly branched pattern of vascular networks, microfluidic lung assist devices can potentially provide more efficient gas exchange and support the metabolic needs of a patient. Hoganson et al. [10] emphasized the importance of utilizing such architectures, compared to straight channels, to enlarge the surface area available and maximize the gas exchange rate. Blood flow was introduced through a dense, branching network of microfluidic channels with a thin gas-permeable membrane separating the channels from an air chamber, representing the alveolar space. Simultaneously, the device architecture was optimized to achieve controllable, low shear stresses within the capillary subunits and designed to eliminate plasma leakage by using only silicone surfaces to come in contact with the blood over the entire wetted area. By examining different blood flow conditions, it was found that a loss in efficiency (i.e., rate of oxygen exchange) occurs when flow rate is increased. Such results are consistent with a previous study [17], showing that full saturation of hemoglobin is 4 times longer within a 25 *μ*m wide capillary, compared to a 10 *μ*m wide one. Considering the fact that Hoganson et al. conducted their experiments in 200 *μ*m wide channels, a loss in efficiency was to be expected. Overall, minimization of channel diameters in parallel to increased membrane permeability can significantly improve gas exchange efficiency within microfluidic lung assists.

Further work by Hoganson et al. [14] addressed some of the considerations for matching physiological flows and preserving shear stresses within the physiological range, while simultaneously mimicking vascular anatomy. The resulting microchannel network design was based on flow optimality rules (i.e., minimization of viscous resistance), known as the Hess-Murray law, and on mimicking an anatomically accurate architecture (in terms of vessel diameters, bifurcation angles, vessel length, and 1 : 1 ratio between height and width of the channel). Moreover, the device was fabricated using micromilling techniques, allowing for higher precision compared to traditional milling or photolithography methods. In spite of these advantages, significantly higher efficiency of gas exchange was not established in this design relative to previous AL devices. However, in view of potential medical use, multilayered devices could be obtained by stacking several functional units (i.e., each unit consists of the blood flow microchannels, an air chamber, and the gas transfer membrane). For such purposes, 115 layers would be required to achieve proper function in a patient; however, such number would drastically enlarge the artificial device and prevent its implantation within the chest cavity.

*μ*FIVDs discussed above all rely on 100% oxygen gas supply. Elimination of such requirement would enable more portability and implantability and help take a leap further towards a long-term suitable AL solution. To this end, a novel design was recently introduced to enable blood oxygenation using ambient air (Figure 2) [9]. The device was designed to function with partial pressure of oxygen in air on the gas side; in parallel, fluidic pressure drop across the artificial capillaries was constrained to physiologic values to allow for further implementation *in vivo*. Similar to the abovementioned microfluidic ALs, the device featured branching networks of air- and blood-flow channels, separated by a thin (15 *μ*m) PDMS membrane. By minimizing both capillary height and membrane thickness, gas exchange efficiency for this AL was found to be comparable with both commercially available and other microfluidic devices [14]. In contrast, since the objective of the study was to maximize surface area rather than to optimize flow conditions, shear stresses within the device exceeded the physiological range and thus increased the risk for RBC damage and thrombus formation.

Without a doubt, the impact of *μ*FIVDs towards engineering an artificial lung is of high value. Although significant improvements are still required both in the design and manufacturing of ALs, microfluidic devices are contributing toward future clinical applications of respiratory support.

## 3. *In Vitro* Models of the Pulmonary Air-Liquid Interface

The luminal surface of the lungs is populated with a confluent, uninterrupted epithelial cell sheet that exists as a continuum across the airway tree, from the larynx down to the alveoli [18]. Airway epithelial cells are covered with an extracellular liquid lining layer, which in combination with air on the luminal side of airways, creates the air-liquid interface (ALI) (Figure 1(a)). While in the conducting airways of the bronchial tree, the liquid film consists of sol and gel layers surmounted by a surfactant layer, in the alveolar region, this multiphase film consists rather of a thin hypophase covered by phospholipid-rich surfactant [19]. The presence of surfactant dramatically reduces surface tension at the air-liquid interface and thus prevents the delicate tissue of the airways from collapsing during expiration and allows alveoli to reopen during the next inspiration. Additionally, lung surfactant films are also known to contribute to innate defense mechanisms [20]. The aqueous liquid layer lining is highly dynamic and is reported to vary from approximately 10 to12 *μ*m in the trachea, to 2.5 *μ*m in the bronchi and finally 0.1 to 0.2 *μ*m in the peripheral airways [19]. For a detailed discussion on properties of the liquid lining layer, including its structure and function, we refer the reader to extended reviews on the topic [21–25]. Below, we elaborate on *in vitro* airway models mimicking physiological aspects of ALI.

Traditionally, *in vitro* experimental setups have been conducted using isolated cells under submerged conditions [26–28]. In these studies, *in vitro *models are composed of cell populations fully immersed in a culture medium. However, in the specific case of pulmonary cells, these submerged *in vitro *conditions only loosely reflect the actual physiological environment characterizing the alveolar epithelium (Figure 1(a)): inside airways, epithelial cells are constantly exposed to ALI. In other words, realizing an experimental setup with cells cultured at an ALI is of critical importance to mimic correctly *in vivo *airway conditions. In particular, *in vitro* culture conditions where the apical side of a cellular monolayer is exposed to air are known to induce cellular differentiation and to cause cells to express morphological and secretory phenotypes matching those found *in vivo *[29]. Of particular importance is the secretion of pulmonary surfactant by type II alveolar epithelial cells [30]. In the case of particle-related drug delivery and cytotoxicity, past studies emphasize the critical role of ALI in determining the characteristics of particle submersion upon depositing on the epithelium [19, 31]. For instance, composition, morphology, and physical-chemical properties of particles are significantly altered when suspended in medium due to interactions with medium components.

**Figure 3:**
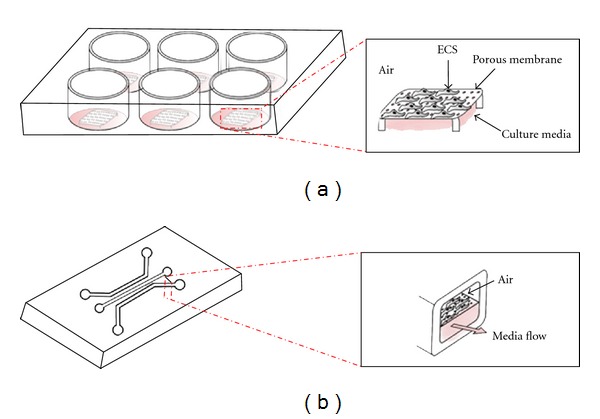
(a) Schematic of a macroscopic *in vitro *model of pulmonary airways recreating an ALI; epithelial monolayers are cultured on a porous membrane in cellular culture inserts to create compartmentalization: apical side of cells is exposed to air while basal side is maintained with growth media. (b) Schematic of microchannels separated by a porous membrane. This setup yields a sandwiched structure allowing for airflow in the upper chamber (i.e., ALI), while maintaining constant perfusion in the lower chamber for cell viability.

To deliver an ALI within a cellular environment, macroscopic *in vitro *models of airways have primarily relied on cell culture inserts in well plates, with membranes permeable to growth media (Figure 3(a)). When epithelial cells are cultured on such membranes, compartmentalization of the cultured cells to each side of the membrane is realized, leading to a separation between conditions set on the apical and basal side of the epithelial monolayer, respectively [30, 32]. Such configuration allows exposure of the upper surface to air (i.e., replicating an airway lumen in an alveolus), while continuously maintaining growth media at the lower surface (i.e., basal side) to support cell viability. Although the abovementioned experimental setups recreate successfully the essence of an ALI, they fail however to replicate the actual cellular microenvironment by neglecting physiological conditions applied on the epithelial monolayer, including respiratory flows on the cells.

To address the limitations of reproducing an accurate pulmonary air-liquid environment with traditional macroscopic approaches (i.e., well plates, petri dishes, etc.), *μ*FIVDs have become increasingly popular in allowing both a precise control of the cellular microenvironment and simulating physiological flow conditions. To the best of our knowledge, Huh et al. introduced the first design of a *μ*FIVD that replicates closely the alveolar air space and created the epithelial air-liquid interface [33]. In their study, two micro-chambers, separated by a porous membrane, were fabricated and small airway epithelial cells (SAECs) were seeded on the porous membrane (Figure 3(b)). This seminal design featured a sandwiched structure, allowing for airflow in the upper chamber in parallel to providing constant perfusion with growth media on the basal side of the epithelial monolayer. In addition to exposure of cells to air leading to differentiation and expression of secretory and morphological phenotypes, the cellular monolayer was exposed to physiological flows of both liquid (on the basal side of the monolayer) and air (on the apical side) in an effort to simulate breathing conditions. Results showed that the air-liquid interface induced SAECs differentiation by expressing Clara cell 10-kDa proteins (CC10), a known marker for differentiated and chemically functional SAECs. In contrast, cells maintained under constant perfusion did not express any CC10 over the period of culturing. Moreover, higher integrity of the cell monolayer was established due to tight junction formation, and it was significantly higher for cells cultured at an ALI in comparison to cells cultured solely under liquid perfusion.

To create a stable and viable ALI over long durations (more than three weeks *in vitro*), parallel efforts have characterized at length growth requirements under constant perfusion for a monolayer of human alveolar epithelial cells (i.e., A549 cell line) in a microfluidic platform [34]. This cell line is widely used as a model for human type II alveolar epithelial cells [35]. In particular, Nalayanda et al. [36] examined cell viability, monolayer integrity, and surfactant production by cells both under submerged conditions and for air-exposed cultures [36]. It was found that epithelial cells cultured at an ALI exhibit higher cell viability and integrity together with decreased surface tension in response to surfactant production, when compared to cells cultured under conventional media exposure. These latter findings are in agreement with Huh et al. [33] as well as recent studies exhibiting increased production of surfactant [37] and higher transepithelial electrical resistance (TEER) [38] for type II epithelial cells maintained at an ALI. Furthermore, A459 cells were found to resist higher mechanical forces (i.e., fluid shear stresses) when cultured at an air-liquid interface. In a very recent study [38], phosphate buffer saline-based (PBS) liquid plugs were propagated upon both submerged and air-exposed epithelial monolayers, creating fluid shear stress upon the cells. Although the epithelial cells at the ALI did not maintain viability, they remained nevertheless attached to the culture substrate even after propagating 10 liquid plugs over it. In contrast, cells cultured under liquid perfusion completely detached from the substrate after no more than 3 propagating plugs.

The above findings emphasize the significance of replicating air-exposed culture conditions and recreating adequately the air-liquid interface to achieve physiologically-realistic conditions within *in vitro* models of small airways and alveoli. *μ*FIVDs offer a viable strategy to integrate these biological constraints and simultaneously provide both anatomically- and physiologically-realistic environments of the respiratory tract.

## 4. Liquid Plugs and Cellular Injury in Pulmonary Airways

In the distal airways of the lung (e.g., <1-2 mm in diameter), physiological changes in the thin liquid film are known to cause acute lung injury and epithelium damage. The origins of such alterations arise from abnormal physical forces, due to mechanical ventilation or fluid shear stresses, as well as from surfactant dysfunction (i.e., disruption of surfactant metabolism) [39]. In particular, modification in cellular functions is intimately linked to pathogenesis and structural remodeling of the lungs, a consequence of respiratory diseases including asthma, chronic obstructive pulmonary disease, cystic fibrosis, and respiratory distress syndrome (RDS) [40–43].

As mentioned earlier, pulmonary surfactant in the alveolated region of the lungs reduces dramatically surface tension at the air-liquid interface, that is, with magnitudes found between near-zero values (at end-expiration) and approximately 25 mN/m (at end-inspiration), well below values for a simple air-water interface, that is, ~70 mN/m [18]. However, insufficiency in surfactant production, or conversely surfactant dysfunction, can not only lead to respiratory impairment and airway collapse, it can also give rise to two-phase, air-liquid instabilities, creating liquid plugs that occlude small airways and impedes gas exchange (Figure 1(a)) [44, 45]. In particular, distension during inspiration of the airway surface can propel the plug further downstream into the airways, ultimately causing plug rupture and airway reopening.

The formation of liquid plugs in small airways can also result from clinical therapies such as mechanical ventilation (MV) and surfactant replacement therapy (SRT) [39]. Yet, these therapeutic strategies are widely used for restoring lung functions. Liquid plugs are also thought to be used as vehicles for targeted drug delivery [46]. Whether a consequence of therapeutic intervention (e.g., MV, SRT, and drug delivery) or physiological disorders (e.g., surfactant dysfunction or insufficiency), the propagation of liquid plugs along respiratory airways, in conjunction with the progression of airway reopening, are believed to produce localized, yet severe fluid mechanical stresses on the underlying epithelial surface [47]. Both experimental and theoretical studies based on *in vivo* animal and computational models, respectively, suggest that abnormal shear stresses developed as a result of fluid mechanical stresses during the airway reopening can potentially injure the epithelial cells and lead to a variety of respiratory complications [47–51].

To explore the fundamental fluid mechanics of liquid plug propagation and its damaging effects on respiratory airway walls, experimental investigations have been initiated through the development of *in vitro* airway models. These efforts include several bench-top models mimicking physiologically-realistic airways and address specifically the characteristics of airway closure and reopening upon the passage of a liquid plug. For instance, thin-walled polyethylene tubes of different radii using lining fluids of different surface tensions and viscosities were used as a model airway to measure the relationship between airway opening velocity and the applied airway reopening pressure [50]. Further, airway models coated with Newtonian lining fluids of constant viscosity were used to investigate a collapsed airway surrounded by parenchyma and characterize how tethering and fluid forces interact to affect the reopening pressure [52]. Eventually, various types of rigid straight and bifurcating tubes, either dry or prewetted, were fabricated to analyze the physical phenomena of liquid plug transport through lung airways [53]. In parallel to these experiments, several computational studies were also conducted to predict the behavior of liquid plugs in airways [49, 51, 54, 55]. For example, a liquid-filled flexible-walled channel, taken as an initial model of pulmonary airway reopening, was developed to investigate the progression of a finger of air through airways closed with liquid plugs [50]. While the abovementioned studies have been successful at examining the physical phenomena characterizing the dynamics of liquid plug transport, they are, however, limited in uncovering the interaction between plugs and the underlying epithelial cell monolayers. These interactions are known for example to give rise to surface-tension-induced epithelial damage during airway reopening [47].

Given such shortcomings, more advanced platforms of idealized model airways have recently surfaced, including a parallel-plate flow chamber that features channels lined with monolayers of fetal rat lung epithelial cells (CCL-149) [47]. There, the reopening of a collapsed airway was mimicked by the progression of a semi-infinite air bubble in a narrow fluid-occluded channel of the chamber. This study revealed that the progression of a semi-infinite bubble in a narrow channel lined with pulmonary epithelial cells induces significant injury to the epithelial cell population and that the addition of pulmonary surfactant significantly alleviates such cellular injury. In another work, the magnitude of pressure, rather than the duration of stress exposure, was found to constitute the major determinant in controlling the degree of cellular injury during airway reopening [56]. Although these seminal studies represent a major advance in investigating experimentally fluid shear stress-induced cellular injury, they come short of mimicking accurately the *in vivo* airway environment. To begin, one of the major differences lies in the lack of mechanical flexibility (i.e., lung tissue distensibility) featured in the experimental models. Moreover, such experimental models are devoid of a collagen substrate beneath the epithelial cells. This latter point is of importance since the pressure exerted during airway reopening could be buffered by collagen proteins, owing to its flexible nature, under *in vivo* conditions [57]. Finally, size and geometry of the model airways significantly differ from actual *in vivo* airway anatomy. Altogether, these limitations and others have motivated researchers to explore new experimental strategies to integrate more physiologically-realistic airway environments.

*μ*FIVDs have shown great potential towards creating *in vitro* airway models that combine the generation and transport of liquid plugs with cell cultures, all integrated within a single platform [33, 39]. Recently, a microfabricated lung airway model featuring two-phase, air-liquid microflows has demonstrated its potential for (i) engineering lung morphologies on a chip and (ii) generating physiological and pathological liquid plug flows while (iii) modeling a physiologically-realistic ALI, as noted earlier [33]. Outcomes from this study confirmed earlier findings that the pressure associated with airway reopening could be injurious to epithelial cells. However, the major finding of this work lies in the critical importance of the final steps occurring during reopening events, that is, the progression of very thin liquid plugs and their rupture in promoting mechanical tissue injury. In the footsteps of Huh et al. [33], a microfluidic lung airway model integrated with a computer-controlled on-chip plug generator and equipped with pressure sensors, solenoid valves and flow meters, was recently fabricated to address the precise control and measurement capability of pressure levels in these microenvironments [38]. This device delivers a solution to obtain reproducible, well-defined liquid plugs, and ultimately enables the analysis of epithelial cell response to shear stress associated with liquid plug propagation under physiologic differential pressures. These recent results indicate that knowledge of local, *in vivo* pressures can provide a better understanding of the fluid shear stress-induced lung injury. Furthermore, *μ*FIVDs have been utilized to investigate the effect of wall flexibility on the plug propagation and the resulting wall stresses in flexible microchannels that closely mimic human small airways [58].

In the past, the development of an *in vitro* network of the airway tree was thought to be a difficult task to perform using conventional “large-scale” approaches. However, *μ*FIVDs have opened the possibility of creating an airway tree network and generating air-liquid interfaces within an integrated single platform [33, 36]. For instance, a simplified microfluidic model of pulmonary airway tree with five generations was recently developed to study the motion of liquid plugs arising at lung bifurcations [59]. While this design did not feature an epithelial cell monolayer, the airway tree model did characterize liquid dispersion along the airway branches. In parallel, a microfluidic alveolar model was recently developed to expose cultured monolayers of alveolar epithelial cells (A549) to combinations of mechanical stresses and surface tension [60]. This study is the first of its kind to study the combined effects of fluid mechanical (i.e., liquid film motion) and solid mechanical stress (i.e., distension-contraction movements) on alveolar epithelial cells. To extend the applicability of microfluidic platforms, an airway model of wound-healing was designed to investigate the regeneration of a wounded lung epithelial cell layer exposed to hepatocyte growth factor [61]. Altogether, these recent microfluidic-driven efforts have laid the foundation for recreating *in vitro* models of lung airways that can be useful for exploring liquid plug propagation, lung injury, and wound healing while circumventing many of the limitations seen in conventional large-scale systems.

## 5. *In Vitro* Models of the Alveolar-Capillary Barrier

The functional efficiency of the lung is primarily characterized by the delicate structure that separates alveolar air from capillary blood (Figure 1(a)). This thin barrier (<1 *μ*m [8]) is comprised of three discrete layers: the alveolar epithelium, the capillary endothelium, and the basement membrane separating the two cellular monolayers [62]. Besides serving as a barricade between the air and blood side, the alveolar-capillary barrier (ACB) is critical for regulating various physiological functions including (i) gas exchange (oxygen and carbon dioxide), (ii) transport of solutes and proteins between capillary blood and alveolar air, (iii) alveolar fluid clearance, and (iv) liquid homeostasis [1, 63]. Since the alveolar lumen surface is susceptible to microbial infection, injury, and inflammation, the ACB also regulates defense mechanisms by controlling the movement of macrophages and lymphocytes from the interstitium and/or capillaries toward the alveolar lumen surface. Alternatively, the diffusive nature of the barrier may be exploited as well for targeted drug and gene delivery [64]. Barrier integrity is mainly dependent on the tightness of the alveolar epithelium and endothelium for the passively transported molecules [1]. Due to the difference in the composition of the interstitium, one side of the alveolar-capillary membrane is found to be thinner than the other, as evidenced by ultrastructural image analysis of the cross section of a capillary lying within the alveolar wall [62]. The thinner part is mainly involved in dynamic gas exchange processes, whereas the thicker part contributes to fluid flux and higher resistance against mechanical hydrostatic pressure. Damage to the barrier, as a result of acute lung injury for instance, can cause alterations in epithelial and endothelial permeability [65, 66], increased inhaled nanoparticle translocation to the systemic circulation [64] as well as pulmonary edema [62] and injury to epithelial cells [65].

With its large surface area and close juxtaposition with underlying capillaries, alveoli are considered the functional units of the lung [67]. Due to their small size (~250 *μ*m) and the large surface to volume ratio available for air, alveoli are inherently unstable structures since surface tension forces at the ALI have a natural tendency to collapse the lung airspace [1]. The thin liquid lining fluid covering the alveolar epithelium isolates it from direct contact with air while reducing potentially harmful effects of surface tension. The underlying epithelium consists of cuboidal type II cells and squamous type I cells. An overwhelming portion of the alveolar surface (>90%) is covered by type I cells since their principal function is to constitute the thin barrier between blood and air. Although the number of type II cells is about twice the number of type I cells, type II cells make up for only about 7% of the alveolar surface. However, their major function is vital: [the secretion of surfactant, a lipoprotein-like substance, via exocytosis of secretory vesicles termed lamellar bodies (LBs).] Surfactant consists of glycerophospholipids (~80%), with dipalmitoylphosphatidylcholine (DpPC) as the predominant component, cholesterol (~10%), and proteins (~10%) including mainly (SP)-A, SP-B, SP-C, and SP-D. Besides, type II cells are also actively involved in the metabolism of xenobiotics, transepithelial movement of water and ions, and the regeneration of type I cells during normal lung development or following lung injury. Alveolar epithelial cells form tight connections with each other by various types of cell-cell contacts, that is, tight junctions, adherence junctions, and others; the anchoring of such contacts is governed by several groups of proteins [1].

While the crucial role of the ACB in various physiological processes has been addressed quite extensively in the past using *in vivo* (e.g., animals and human models) approaches [68–70], developing an *in vitro* model system that can mimic the *in vivo* barrier conditions remains a challenging task. The need for developing such model platforms is motivated by the intricate and delicate nature of alveoli that creates hurdles in understanding cell-cell interactions between different cell types, as well as answering other cell-biology-related questions with reference to the physical barrier itself [1]. In addition, considerations for the ACB as a potential route for systemic delivery of therapeutic drugs, or alternatively as a route for inhaled toxic (nano)particulate matter to access the systemic circulation, call for *in vitro* ACB models to study quantitatively both (i) drug absorption phenomena and (ii) aerosol translocation processes across the ACB. Although *ex vivo* approaches (i.e., using excised lung tissues) have been considered useful models for examining lung injury, barrier permeability, and alveolar fluid clearance, the precise determination of cellular processes leading to barrier permeability, particle translocation, and drug absorption mechanisms lies widely out of reach using such methods. Therefore, there is a need for convenient, reliable, and robust *in vitro* cell-based models that not only possess a realistic ACB anatomy, but can also facilitate the study of such physiological processes.

A growing number of cell lines derived from different parts of the airways are available for *in vitro* ACB studies [1]. Examples include human type II alveolar epithelial cells (A549), human bronchiolar epithelial cells (NCI H-441), rat type-I-like alveolar epithelial cells (R3/1), rat type-II alveolar epithelial cells (L-2), and mouse type-II alveolar epithelial cells (MLE-12 and 15). Among these, the A549 (American Type Culture Collection, ATCC CL-185) line is the most frequently used epithelial model that is immortalized and derived from human lung adenocarcinoma [35]. The morphology and biochemical features of this cell line resemble the characteristics of human pulmonary type II cells *in situ*. However, the major problem with A549 cells is their incapacity of forming functional tight junctions, that is, tight monolayers of polarized cells. Therefore, primary cultures of lung epithelial cells, in conjunction with pulmonary microvascular endothelial cells (PMEC), are recommended in order to reconstruct a more complete alveolar-capillary barrier. Since there are limited sources of human lung tissues, as well as ethical considerations related to the use of human lung and pulmonary microvascular tissues, the majority of studies have been conducted on cells isolated from the lungs of animals, including rat, mouse, rabbit and pig [1]. However, the physiological relevance of the data stemming from such animal models may not be necessarily accurate when considering the specific human system, as the precise ACB structure and the actual pathway for physiological processes differ significantly among species [71].

As a result, several research groups have established *in vitro* cellular models using instead lung epithelial cells and endothelial cells of human origin. For instance, a coculture system of human distal lung epithelial cells (NCI H441) and human pulmonary microvascular endothelial cells (HPMEC) using a filter membrane was developed to study cellular interactions of the ACB epithelium and endothelium in both pathogenesis and recovery from acute lung injury [72]. A comparable model system has also been used for predicting effects of novel drugs on healthy or inflamed tissues [64]. Similarly, to examine pathomechanisms occurring at the ACB during acute lung injury, a primary coculture system was established by cultivating HPMECs with primary isolated human type II alveolar epithelial cells (HATII) on opposite sides of a permeable filter support [73]. Most recently, a model made of an epithelial cell line (H441) and an endothelial cell line (ISO-HAS-1) of human origin was used to examine the inflammatory and cytotoxic responses of monodisperse amorphous silica nanoparticles (30 nm) at the ACB [74]. In spite of such progress, the abovementioned model systems do not incorporate yet other cell types such as macrophages, lymphocytes, dendritic cells, and polymorphonuclear cells that characterize a true ACB. Moreover, these platforms lack the ability to recreate the mechanical properties of the interface (e.g., wall distensibility).

*In vitro* cellular models based on microfluidic technology have been envisioned to address the limitations of traditional coculture approaches and mimic the structural, functional, and mechanical properties of human ACB. For example, a multistage *μ*FIVD integrated with a suspended porous membrane was recently designed in order to recreate the alveolar-capillary membrane [75]. The functional characteristics of the device were validated by the culturing of human pulmonary endothelial (HMEC-1) and alveolar epithelial (A549) cell lines within the device. Although this work represents a step towards recreating a preliminary model of ACB, the specific model lacks the *in vivo* structure of ACB, such as coculturing pulmonary epithelial and endothelial cells on opposite sides of the membrane. Despite such limitations, the microfluidic study characterized optimal growth conditions for alveolar epithelial cells cultured at ALI. Most recently, a more sophisticated microfluidic-based model was designed to mimic the critical functional aspects of human ACB [37] and simultaneously implement wall expansion/contraction to replicate breathing movements. This microfluidic solution is achieved by culturing human AECs and human PMECs in two closely apposed microchannels separated by a porous and flexible PDMS membrane. By incorporating lateral microchambers on both sides of the ACB, subatmospheric pressure-driven stretching was recreated upon applying vacuum. This model system reproduces complex integrated organ-level responses to bacteria and inflammatory cytokines introduced into the alveolar space.

The pioneering microfluidic ACB model of Huh et al. [37] was also found to be useful for nanotoxicological research. Namely, cytotoxicity effects of toxic nanoparticles exposed at ALI were addressed. The *μ*FIVD revealed that cyclic mechanical strain accentuates toxic and inflammatory responses of the luminal surface to silica nanoparticles. Alveolar epithelial cells exposed to ultrafine (12 nm) silica nanoparticles in the absence of mechanical strain showed little or no reactive oxygen species (ROS) production; in contrast, the intracellular formation of ROS was significantly increased in the presence of mechanical distortion. Similarly, the intracellular levels of ROS in the underlying endothelium were also found to be significantly elevated in the presence of cyclic strain. Moreover, mechanical strain enhances epithelial and endothelial uptake of nanoparticulates and stimulates their transport into the underlying microvascular channel (i.e., translocation).

## 6. Conclusions and Future Directions

Developing anatomically and physiologically accurate models of the human respiratory system is of high importance towards applications for lung assist development as well as cytotoxicity and lung injury studies at the alveolar level. For these purposes, microfluidic systems hold great potential for use as a platform in *in vitro* pulmonary airway studies; *μ*FIVDs are able to successfully replicate the delicate structure of the native lung while incorporating critical respiratory functions. The novel technologies reviewed here have deeply contributed towards understanding cellular behavior and physiological processes at the alveolar level. While the use of *μ*FIVDs is rapidly rising in popularity, these platforms will need, in the future, to incorporate additional cell types (i.e., macrophages, dendritic cells, etc.) in an effort to mimic regulatory and immune processes characteristic of the alveolar-capillary barrier. In addition, although significant research is still required before clinical applications of an artificial lung can be commercially available and replace current technologies, microfluidic systems have shown feasibility and demonstrated excessive advancement in the efficiency of artificial respiratory support. Overall, respiratory physiology on a chip is a noteworthy technology towards research and development of clinical applications in human respiratory disciplines.

## Figures and Tables

**Table 1:** Specific advantages and limitations of *in vitro* models of the respiratory environment.

	*In vitro* devices	Advantages	Limitations
Artificial lung	Conventional	(i) Commercially available (ii) Widely used technology in patients	(i) Low gas exchange rates (ii) Mobility restrictions
	Microfluidics	(i) Mimics lung anatomy (ii) Miniaturized and portable	(i) Not yet clinically applicable (ii) No product available on the market (iii) Requires complex designs

Air-Liquid interface	Conventional	(i) Open access (ii) Easy to probe and measure	(i) No physiological flow
	Microfluidics	(i) Captures alveolar architecture (ii) Mimics physiological flows	(i) Immune testing studies yet to be accomplished (ii) Mechanical stretching requires sophisticated design

Liquid plugs	Conventional	(i) Easily adaptable	(i) Static (inflexible) system
	Microfluidics	(i) Automated and controlled system (ii) Anatomically realistic airways (iii) State-of-the-art platform for airway reopening studies	(i) Requires high technical design and fabrication skills

Alveolar-capillary barrier	Conventional	(i) Easy and convenient to culture and maintain	(i) Static culture conditions (ii) No continuous waste removal
	Microfluidics	(i) Mimics physiological (air/blood) flow environment (ii) Permits mechanical stretching (iii) Physiological length scales and ratio of cell volume to extracellular fluid	(i) Sophisticated culturing processes
